# Is Participation in After-School Physical Activity Associated with Increased Total Physical Activity? A Study of High School Pupils in the Czech Republic

**DOI:** 10.3390/ijerph7072853

**Published:** 2010-07-13

**Authors:** Jana Pelclová, Walid El Ansari, Jana Vašíčková

**Affiliations:** 1 Center for Kinanthropology Research, Faculty of Physical Culture, Palacky University, Tr. Miru 115, Olomouc 77111, Czech Republic; E-Mails: jana.pelclova@upol.cz (J.P.); jana.vasickova@upol.cz (J.V.); 2 Faculty of Sport, Health and Social Care, University of Gloucestershire, Oxstalls Campus, Oxstalls Lane, Gloucester GL2 9HW, UK

**Keywords:** pedometer, adolescents, self-determined physical activity, day, month and season variability, physical education

## Abstract

This study assessed the physical activity (PA) levels and its variability across days, months and seasons of two groups of high school pupils: those who did and those who did not participate in regular organized after-school physical activity (ASPA). Thirteen pupils wore pedometers continuously for one school-year, logged their step counts into record sheets and were then interviewed for information as regards their participation in any ASPA. Repeated measures analysis of variance showed that regardless of the day, month and season, ASPA pupils achieved significantly more mean step counts/day than the non-ASPA pupils. There were no significant fluctuations across months and seasons in PA levels of ASPA pupils when compared to non-ASPA pupils. We conclude that regular organised ASPA might increase the pupils’ total PA levels; and could help to maintain a relatively constant PA level for adolescents across the whole school-year regardless of the influences of a range of weather and meteorological indicators that are related to months/seasons.

## 1. Introduction

The health benefits of regular PA in youth include the prevention of many risk factors (obesity, high levels of body fat and cholesterol, hypertension) that could set up themselves throughout childhood and project into adulthood [1,2]. PA in childhood/adolescence seems also linked to educational benefits. Swedish pupils’ academic achievement was associated with PA and fitness [3], and in the USA, increasing the opportunities for PA for fourth, sixth and eighth grade pupils supported academic achievement even after controlling for pupils’ weight, ethnicity, gender, grade, and socioeconomic status [4].

Children currently expend ≈525 kcal/day less than their counterparts 50 years ago [5]. Further, PA drops exponentially during the adolescent period [6], and many adolescents place their health at risk due to insufficient PA [7]. Indeed, studies in the Czech Republic showed a decline of PA from the preschool period to pupils 12 years old and moreover, between 12–18 years [8,9]. Such findings have stimulated proposals about the ways by which young people could enhance their PA levels. However, although adolescent PA patterns may differ by time of day, little is known about after-school PA (ASPA) behaviours [10]. This is despite the fact that the after school period is a critical interval that defines the propensity of youth for PA [11]. After-school hours may represent significant opportunities for educators and researchers to modify adolescents’ PA behaviours. Hence it is important to understand the patterns of adolescents’ PA (during the school and AS periods) and its natural variability across the year in order to guide effective PA interventions.

When compared with the school period of the day, AS periods represent a ‘critical window’ for youth to accumulate necessary health-related PA. In the Czech Republic, 104 preschoolers (aged 5–7 years), 1174 teenagers (aged 12–17 years) and 787 young adults (aged 18–24 years) had approximately three times lower activity energy expenditure during school time compared to the out of school time periods [8]. Likewise in New Zealand, high schools pupils (12–18 year old) exhibited substantially lower participation levels in PA during school hours (at recess and lunchtime) than for the time periods that were outside of the school hours (before and after school) [12]. Similarly, for both genders, the pedometer-measured steps of British children (7–11 year old) suggested that steps accumulated in weekday leisure time were greater in the high-active groups than in the mid- and low-active groups, with relatively smaller differences between the activity tertiles for steps accumulated at school [13]. In northwest England, others have reported that the accelerometer-measured activity of 58 children (aged 7–11 years, monitored on 4 consecutive weekdays) was conversely lower in volume and similarly less stable after school day had ended [14]. These findings reinforce the importance of ASPA, especially organized sports activities [15]. Indeed, in the U.S.A, youth who participated in organized ASPA achieved significantly more steps/day than their non-participating counterparts [16].

In spite of the limited evidence suggesting that AS programs can improve health-related aspects [17], there seems consensus that the AS period should be targeted for PA interventions [16], probably because AS hours appear to have a great potential for increasing PA levels, which augment a child’s total daily energy expenditure [16]. Although PA interventions during the school day hold great prospective and remain important, ASPA programs are emerging as potentially useful and feasible for PA promotion [17].

Nevertheless, few studies have examined the differences in PA patterns between regular organized ASPA participants and non-ASPA participants. E.g., Flohr *et al.* [16] evaluated the pedometer-assessed PA patterns in children and assessed differences of PA levels in ASPA participants and non-participants for two consecutive weeks; Santos *et al.* [15] used a questionnaire and assessed the relationship between participation in organized and non-organized PA, commuting to school and BMI; and, Beets *et al.* [17] meta-analyzed the impact of after-school programs on PA and fitness. To the best of our knowledge, there seems no research that investigated regular ASPA participation across longer periods of time e.g., the school-year, thus allowing the examination of its effects across days, months and seasons.

### 1.1. Aims of the Study

Therefore, the aim of the present case study was to monitor the pedometer-determined PA over a school-year for two groups of high school pupils: those engaged in regular organized after-school physical activity (ASPA); and those who were not engaged in regular organized after-school physical activity (non-ASPA) in the Czech Republic. The three specific objectives were to:

Measure and appraise the pedometer-determined PA by day (weekdays/weekends; provision/non provision of physical education lessons at school), month and season for the two groups of pupils; Compare the pedometer-determined PA levels by day, month and season for the two groups of pupils; and,

Assess the variability in pedometer-determined PA by day, month and season for the two groups of pupils.

## 2. Methods

### 2.1. Participants

This longitudinal study was approved by Palacky University (Czech Republic). A high school in Olomouc city (Moravia region, Czech Republic) was selected where the study was implemented, premised on previous cooperation between the Center of Kinanthropology Research at Palacky University and the school’s physical education (PE) teachers. Such history of successful past collaboration was important, as effort was required for supervising the use of pedometers across the whole year of the research, as well as motivating and assisting the participating pupils (e.g., adjusting the pedometers, changing the batteries, *etc.*). In line with others [18], the selection criterion for pupils to participate in the study was that they were ambulatory and willing to monitor their PA behaviour for one year. All pupils were informed of the study objectives. No incentives were offered to participants, the participating pupils and their parents provided a written consent, and pupils could withdraw from the study anytime if they wished.

After receiving permission from the school, the first author approached two of the four grade 1 classes (each class ≈30 pupils) of the high school in Olomouc city at the beginning of each monitored year. Only two of the four classes were approached each year as these two classes (≈60 pupils) had the same PE teacher who had agreed to support the participating pupils during the monitoring process and also provide the pedometer services mentioned above. Of the 120 pupils approached across the two years, 29 actually agreed to participate and started with the monitoring. However 16 pupils dropped out of the study: some did not wish to complete the one-year monitoring (11 pupils dropped out within 1–2 months of the beginning of the monitoring); whilst another 5 pupils did not appear to wear the pedometer regularly (several breaks in the continuity of the pedometer data, each of a duration of >1 week). Hence only 13 pupils (eleven girls and two boys; mean age 15.6 ± 0.5 years; BMI 21.25 ± 2.46 kg/m^2^—data from the beginning of the research) completed the pedometer self-monitoring across the duration of the school year. Of the 13 participants, eight pupils (mean age 16.1 ± 0.6 years; BMI 23.03 ± 2.08 kg/m^2^) were not engaged in any organized ASPA, whereas five participants (mean age 15.8 ± 0.8 years; BMI 19.79 ± 2.39 kg/m^2^) regularly participated in organized ASPA (floorball players) through the whole year that was monitored.

### 2.2. Measures and Procedures

Each of the thirteen pupils wore a pedometer (Omron HJ-105) firmly on the right side of the waist continuously for one year for at least 10 hours per day (except for sleeping, hygiene and bathing). Moreover, participants also logged the number of steps into record sheets that were provided by the research team; and reset the pedometer to zero every morning. At the end of the monitoring year, each participant was also interviewed. The interview questions concerning the ASPA were: a) Did you participate regularly in any organized ASPA across the whole monitored year? and, b) If yes, what kind of activity did you participate in? We also expected differences in pupils’ PA between the days with PE lessons and days without PE lessons [19], so the school teachers provided us with the school’s timetables in order to identify the days that included PE lessons.

The same procedures were applied in monitoring the sample of 13 pupils over the period of two academic years (2 cohorts of participants, each cohort monitored for one year). Hence, nine pupils were monitored for 12 months (16 November 2005–15 November 2006); and another 4 pupils for 12 months (16 November 2007–15 November 2008). There were missing data during the summer holiday (July and August) where pupils’ daily programs in the summer holiday differed from their programs during the academic year as they are not supervised by teachers. Hence in order to avoid bias that could be introduced by including these two holiday months, we analyzed only 10 months of each academic year.

### 2.3. Statistical Analysis

SPSS version 17.0 was used (SPSS, Inc., Chicago, IL) to process the data. Significance level was set at *p* < 0.05. We collected step values data for 3,234 person-days, which is 82.1% of possible 3,939 person-days (if each pupil would have recorded 303 days of monitoring) *i.e.*, 10 months of the school year. In agreement with Tudor-Locke *et al.* [18], missing step values from our dataset were estimated using the Missing Values Analysis EM function of SPSS. Three variables were used to gauge the missing step values for each pupil: the pupil’s unique identifier; day of the week; and month where a given date was missing. About 17.9% of data were supplemented with average values of steps.

The vernal/autumnal equinoxes and the summer/winter solstices marked the seasons: autumn (23 September–20 December); winter (21 December–20 March); spring (21 March–20 June); and summer (21–30 June and 1–22 September). The corrected data (number of steps/day) were analyzed for descriptive statistics (M ± SD, 95% confidence intervals) for the 10 months of the school year: 16 November 2005–15 November 2006; and, 16 November 2007–15 November 2008 (excluding July and August). The number of steps/day for each pupil was compared by day of week; by days with PE classes and days without PE; by month; and by season. Moreover, the participants were divided into two groups according to their engagement or non-engagement in regular organized ASPA.

The variability in pedometer-determined PA by day, month and season for the two groups of participants (ASPA and non-ASPA pupils) was assessed by repeated measures analysis of variance (ANOVA) applied to the corrected data. When there were significant differences between >3 categories of any given variable (e.g., four seasons), Bonferonni post hoc pairwise comparisons were used with statistical significance level set at *p* < 0.001. Repeated measures analysis of variance (ANOVA) also assessed differences in days with/without PE classes and weekends, days of the week, months and seasons between the ASPA pupils (regularly participated in organized after-school PA) and the non-ASPA counterparts (pupils who did not participate in organized after-school PA).

During the initial data analysis we screened for outliers. The data revealed that one of our 13 participants (a girl) who completed the monitoring had a high daily step count across the monitored school year (27,772), which was higher than the means of ASPA and non-ASPA pupils (18,008 ± 8316; 14,306 ± 6,658 respectively). The published literature seems not to offer an agreed consensus on the processing of outliers, hence after discussions between the authors about whether to exclude the outlier data from the current analysis (possibility that it introduces bias), it was decided to retain this outlier in the analysis. On the one hand, analyses that include data that are uncommonly large/small compared to the rest of the data set risk estimating models that are not representative or that introduce variability. On the other hand, analyses that exclude these values without testing their significance as outliers may seriously bias a model [20]. Outliers can help to maximise variation across predictor and outcome variables [21]. Conversely, outliers can have negative effects in statistical models and are commonly deleted/transformed [22,23]. However, Altman [24] contends that if outlying values are not due to a mistake *i.e.*, a data entry error, and the value is plausible it should be left unchanged. Indeed, there seems no clear set of statistical tools/tests available to find an outlier’s significance [20]. In line with Altman [24], the research team felt that the step count values of our outlier were: (a) conceivable; and, (b) not the result of a data entry error. Hence we left it unaltered.

## 3. Results

**Figure 1:**
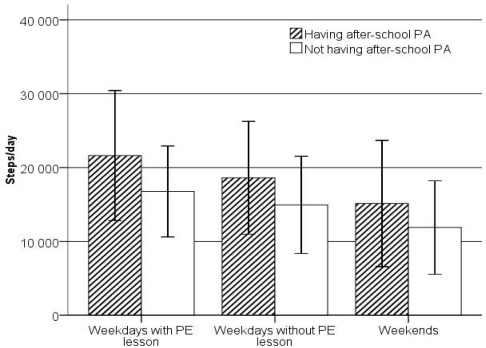
Steps per day (*M*; 95% CI) achieved by ASPA and non-ASPA pupils by weekdays with PE lesson, weekdays without PE lesson and weekends.

ASPA pupils accumulated significantly higher number of steps/day (18,008 ± 8,316) than their non-ASPA counterparts (14,306 ± 6658). These increases in the number of steps/day were evident on weekdays with PE lesson (F = 51.28, *p* < 0.0001), weekdays without PE lesson (F = 151.63, *p* < 0.0001) and on weekends (F = 52.86, *p* < 0.0001) (Figure 1). A step-wise decrease in number of steps/day across weekdays with PE lesson, weekdays without PE lesson, and weekends was evident for both groups of pupils. The differences in steps/day across the weekdays with PE lesson, weekdays without PE lesson and weekends were significant in ASPA (F = 48.77, *p* < 0.0001) and non-ASPA pupils (F = 77.87, *p* < 0.0001).

**Figure 2:**
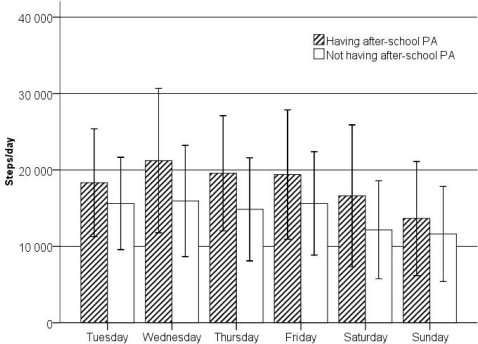
Steps per day (M; 95% CI) achieved by ASPA and non-ASPA pupils by days of the week.

There was significant variability in mean steps achieved on each day of the week in ASPA (F = 20.25, *p* < 0.0001) and non-ASPA pupils (F = 25.02, *p* < 0.0001) (Figure 2). The differences in steps/day between the two groups of pupils were significant on each day of the week (F_Monday_ = 32.06, *p* < 0.0001; F_Tuesday_ = 23.55, *p* < 0.0001; F_Wednesday_ = 55.19, *p* < 0.0001; F_Thursday_ = 60.16, *p* < 0.0001; F_Friday_ = 33.99, *p* < 0.0001; F_Saturday_ = 44.78, *p* < 0.0001; F_Sunday_ = 12.15, *p* = 0.001). The lowest mean step counts were on Sundays regardless of engagement in ASPA (13,653 ± 7,467 for ASPA pupils; 11,613 ± 6,203 steps/day for the non-ASPA pupils).

**Figure 3:**
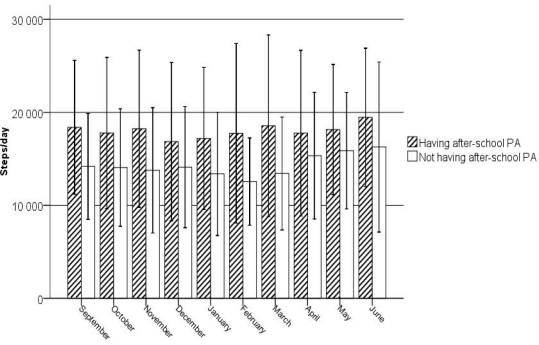
Steps per day (*M*; 95% CI) achieved by ASPA and non-ASPA pupils by months of the school year.

Figure 3 shows that across the different months, there were significant differences in the steps achieved per day in non-ASPA pupils (F = 7.67, *p* < 0.0001) (Figure 3). However, in ASPA pupils, no significant differences were found across the different months (F = 1.18, *p* = 0.306). Regardless of the month of the school year, ASPA pupils had significantly higher mean step counts/day than the non-ASPA pupils (F_September_ = 40.00, *p* < 0.0001; F_October_ = 26.27, *p* < 0.0001; F_November_ = 33.47, *p* < 0.0001; F_December_ = 13.31, *p* < 0.0001; F_January_ = 27.85, *p* < 0.0001; F_February_ = 47.50, *p* < 0.0001; F_March_ = 42.30, *p* < 0.0001; F_April_ = 9.18, *p* = 0.003; F_May_ = 11.40, *p* = 0.001; F_June_ = 12.81, *p* < 0.0001). For ASPA and non-ASPA pupils, the highest mean step counts were in June (19,464 ± 7,433 and 16,273 ± 9,146 respectively). However, the lowest mean steps counts were in December for ASPA pupils (16,856 ± 8,506), whereas it was in February for non-ASPA pupils (12,558 ± 4,686).

**Figure 4:**
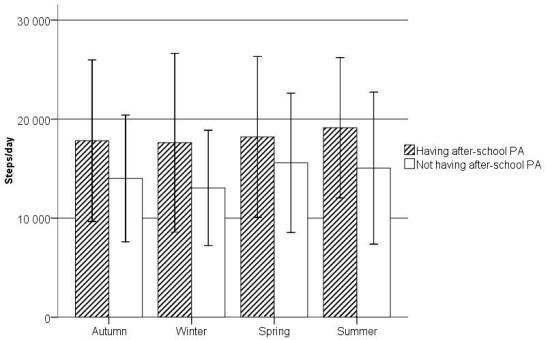
Steps per day (M; 95% CI) achieved by ASPA and non-ASPA pupils by season of the school year.

Figure 4 illustrates the variability in the mean steps/day by season in both groups that were examined. A point to note is that the significant differences in the mean steps/day across all seasons were found only in non-ASPA pupils (F = 19.52, *p* < 0.0001) compared to the ASPA pupils (F = 1.42, *p* = 0.235). In non-ASPA pupils, the post hoc pairwise comparisons showed differences between summer and winter (*p* < 0.001), autumn and winter (*p* = 0.035), spring and winter (*p* < 0.001), and spring and autumn (*p* < 0.001). Regardless of the season, non-ASPA pupils had significantly lower mean steps/day than the ASPA pupils (F_Spring_ = 35.60, *p* = 0.001; F_Summer_ = 28.57, *p* < 0.001; F_Autumn_ = 78.43, *p* < 0.001; and F_Winter_ = 111.19, *p* < 0.001). The biggest difference in mean steps/day between the two groups was in winter (4,571 steps/day), whereas the least difference was in spring (2,613 steps/day).

## 4. Discussion

To the best of our knowledge, the study described in this paper could be the first to pedometer-assess adolescents’ PA levels continuously for the duration of one school year. The implication is that there existed very few studies to compare our findings with. For instance, when our sample (young adolescents) was comparable with other research, the duration of monitoring was different. E.g., only one previous study of 44 young adolescents [16] reported patterns of pedometer-assessed steps/day that included weekend days, physical education days *vs.* non-physical education days, and the contribution of ASPA participation. However, Flohr *et al.* [16] monitored their participants only for 2 weeks (hence their data were not analyzed by season and month). Likewise, when our period of monitoring (1 year duration) was comparable with other research, the sample was not directly comparable. For instance, only one previous study of 23 adults (mean age = 38 ± 9.9 years) monitored pedometer PA for one year [18]. Studies that actually monitored schoolchildren’s PA across different seasons did so for much shorter and interrupted periods of time than the current study undertook e.g., 2 weekends and 2 weekdays days across three seasons (1,115 Auckland children) [25], or 4 weekdays across 2 seasons (256 children in Greece) [26]. Hence, the current study could be a pioneer in describing the continuous long-term day-to-day patterns and variability of pedometer steps/day in relation to pupils’ ASPA or its lack thereof.

As regards the first objective of this study, we appraised the pedometer-assessed PA levels by day (weekdays/weekends; provision/non provision of PE lessons at school), month and season in the two groups of adolescent pupils. On average, our ASPA pupils accumulated significantly higher number of steps/day (18,008 ± 8316) than their non-ASPA counterparts (14,306 ± 6,658). An encouraging point is that both these step count levels compared favourably with levels from a recent review of 31 studies published since 1999 of pedometer-determined PA in youth that reported comparable weekdays (≈12,000–16,000_boys_ steps/day; 10,000–14,000_girls_) and weekend values (≈12,000–13,000_boys_ steps/day; 10,000–12,000_girls_) [27]. Our findings also compared favourably with levels reported from a 13-country review suggesting that boys and girls from European and Western Pacific countries were more active than young people from U.S. and Canada [28]. Furthermore, regardless of attending ASPA, our whole samples’ weekday and weekend values (16,761 ± 7358 and 13,134 ± 7557 steps/day respectively) were also in support of the levels reported by Tudor-Locke *et al.* [27]. Similarly, the weekend values for our non-ASPA and ASPA pupils (11,886 ± 6,325; and 15,131 ± 8,548 steps/day respectively) were correspondingly in agreement with other studies [27]. Moreover, our ≈3,000 step/day difference between weekdays and weekend values were comparable to a study of 64 Polish adolescent using pedometers for three weeks [29].

In connection with objective two, we compared the pedometer-determined PA levels by day, month and season for ASPA and non-ASPA pupils. Adolescents who regularly attended organized ASPA achieved consistently more step/day than those who did not. Moreover, this was true regardless of the ‘type’ of day: *i.e.,* ASPA participants recorded more step counts on all days of week; on days with PE lesson; on days without PE lesson; and also on weekends. In the U.S.A, Flohr *et al.* [16] reported similar findings (44 seventh-grade adolescents, mean age 12.4 years), where their ASPA participants achieved 24% more steps on all days as compared to non-ASPA pupils. In agreement with Flohr *et al.* [16], our ASPA participants logged about 21% more step/day counts than non-ASPA pupils. To our knowledge, no previous study has described pedometer-assessed PA in adolescents who participated in ASPA and those who did not analysed by month and season of the year. Our finding that ASPA participants accumulated grater total volume of pedometer-determined PA than non-ASPA regardless of month and season has implications for long-term PA promotion strategies.

Regarding objective three, the present study assessed the variability in pedometer-determined PA by day, month and season for ASPA and non-ASPA pupils. Few studies analysed the natural variability of PA across substantially long periods of time. Tudor-Locke *et al.* [18] collected 365 days of continuous self-monitored pedometer data (23 adults) to explore the natural variability of PA by month, season, weekday *versus* weekend day, and sport/exercise participation day *versus* non-participation day. However, there seems no previous study of young adolescents that assessed the variability of daily step counts associated with regular participation in organized ASPA analysed by day, month and season.

As regards PA variability by day, we found that the number of daily steps significantly differed by day of the week (weekdays/weekends; provision/non provision of school PE lesson) in both ASPA and non-ASPA participants. This is in support of a study in adults [18] where the day-to-day variability in steps/day corresponded to expected fluctuations with classification of the day (weekday *versus* weekend day, and sport/exercise participation day *versus* non-participation day). Our findings also support research of older adults [30] in which the day-to-day PA variability noticeably reflected the expected oscillation with classification of day (*i.e.*, PA in scheduled exercising weekdays > non-exercise weekdays > weekend days). However our finding of the variability of PA by day is in contrast with Flohr *et al.* [16] who reported that the number of steps/day of young adolescents was consistent across classification of day (*i.e.*, physical education *vs.* health education days *vs.* weekend days). A potential explanation for the lack of PA variability by day reported by Flohr *et al.* [16] is that they monitored their participants for a relatively short duration (only for 2 weeks) in one season. This suggested that for variability in PA to be detected in young adolescents, a longer period of monitoring would be recommended, in contrast to older adults where shorter periods (9 days) of monitoring were able detect variability in PA [30].

As to the PA variability by month and season, whereas daily steps significantly differed by day of the week in ASPA and non-ASPA participants, significant month and season variability was found only in non-ASPA pupils. This is an important finding of the present study: in non-ASPA pupils, PA decreased in winter months and increased in spring, suggesting that activity levels of non-ASPA pupils may be influenced by exogenous factors (e.g., such as ambient temperature, rainfall, snowfall, day length *etc.*). Previous research supported such a proposition: adult weekly leisure-time energy expenditure was ≈15–20% higher during spring and summer [31]. Similarly, primary school children achieved less mean total daily steps counts in winter than in summer [25]. Indeed short day length and extremes of ambient temperature could hinder regular participation in outdoor PA [32,33]. Contrary to our non-ASPA pupils, the differences across months and seasons were not significant in ASPA pupils, suggesting that regular ASPA might contribute to reduce, on achieved PA levels, the possible influence of meteorological (snowfall, wind, hours of sunshine) and winter severity indicators related to months/seasons. Thus, despite our study’s small sample size, the findings suggested the lack of variability in PA levels by month and season for ASPA pupils. For adolescents’ PA behaviour, this could translate into that ASPA participation appears to be a way to maintaining a constant level of PA for adolescents across the whole duration of the school-year.

Collectively, these findings suggested the need for more research into the effects of ASPA, especially organized sports activities [15]. There have been proposals that the potential of after-school hours in increasing children’s PA levels may help to augment a child’s total daily energy expenditure [16].

Although the current study is unique in terms of day, month and season variability in relation to AS participation, it has limitations. The small sample size, probably due to the burden of continuous long-term (one year) self-monitoring, does not allow generalizations. Further, with a larger (and more representative) sample, the external validity of the findings would be enhanced, and any possible gender effects that underlie the group comparisons could have also been dis-entangled. Relatively high steps/day values across the whole school-year in ASPA and non-ASPA pupils suggested that those pupils who self selected themselves and completed the monitoring across a long time period might have been motivated to walk more. The detailed schedule of the type, duration and weekly frequency of participation in organised ASPA would have helped to better assess the nature and particular features of the associations of ASPA. For instance, a comprehensive outline of the number of steps that participants accumulated during the daily segments (during ASPA or PE) would have provided deeper insights into the distribution of the pupils’ step counts across the time periods of the day. In general, the findings of our study are preliminary and require further investigation. Future research into the effects of regular organised ASPA should consider the limitations highlighted above, and would benefit from recruiting more pupils from different settings that would allow gender comparisons. Studies will also need to document the fine characteristics (e.g., nature, degree, volume, intensity, *etc.*) of the ASPA in order to enable a more detailed analysis of the associations under investigation. Finally a detailed log of the pedometer wear-time for each participant would have allowed comparing and controlling for any differences in the periods when participants actually had their pedometers on them.

## 5. Conclusions

In spite of the above limitations, this study could be the first to report adolescents’ patterns of pedometer-monitored steps/day that were assessed continuously across the duration of the school year.

In summary, the study findings suggested that month and season variability of PA differed in ASPA and non-ASPA participants. However, the day-to-day variability of PA levels was similar in ASPA and non-ASPA participants implying the fluctuation of daily PA according to the day of the week (weekdays/weekends; provision/non provision of school PE lesson). Hence, on days with a PE lesson, the mean step counts increased regardless of whether pupils participated or did not participate in ASPA. Therefore, incorporating PE lessons several times per week seems to be warranted and perhaps even more so for those who do not participate in ASPA.

Furthermore, the lack of variability in PA levels for ASPA pupils by month and season in comparison with non-ASPA pupils is a key finding. It suggested that in relation to adolescents’ PA behaviour, regular ASPA might help to reduce the possible influences of a range of meteorological indicators related to months/seasons; and that regular organised ASPA seems to be a way to maintaining a relatively constant PA level for adolescents across the whole school-year.
